# Effects of Common Biochar and Acid-Modified Biochar on Growth and Quality of Spinach in Coastal Saline Soils

**DOI:** 10.3390/plants12183232

**Published:** 2023-09-11

**Authors:** Juan Wang, Danyi Shi, Chengzhen Huang, Biyu Zhai, Shaoyuan Feng

**Affiliations:** College of Hydraulic Science and Engineering, Yangzhou University, Yangzhou 225009, China; mz120211074@stu.yzu.edu.cn (D.S.); MZ120221136@stu.yzu.edu.cn (B.Z.)

**Keywords:** biochar addition, acid-modified biochar, photosynthetic characteristics, quality, water productivity

## Abstract

The rational development and efficient utilization of saline soils can alleviate the problem of insufficient arable land faced by agricultural production in China. A prominent problem is improving soil salt and water conditions for promoting land resources’ productivity in coastal areas. Biochar is widely used for soil improvement, as it has remarkable properties. A pot experiment was conducted to study the effects of two kinds of biochar (common biochar and acid-modified biochar) with three addition rates (2%, 4%, and 8%) on the growth, yield, photosynthetic characteristics, and quality of spinach. The results revealed that 2% and 4% common biochar increased the plant height, stem diameter, and leaf area index, effectively improving the yield of spinach and water productivity, while 8% common biochar was detrimental to the growth of spinach to some extent. Acid-modified biochar significantly benefited the growth and increased the water productivity of spinach, ensuring high yields, while also improved quality. Similarly, acid-modified biochar was less effective at high additions than at low-to-medium additions. The integrated biological response version 2 (IBRV2) values under acid-modified biochar treatments were all significantly higher than those under common biochar, but there is no significant difference among three treatments in the same biochar group, which suggested a pronounced amelioration in spinach growth within saline-alkali soil upon the incorporation of acid-modified biochar. Overall, applying acid-modified biochar at the rate of 4% exhibited enormous potential for increasing the yield and quality of spinach in saline soils.

## 1. Introduction

It is reported in the third National Land Survey that the cultivated land in China had decreased at an average rate exceeding 0.67 million ha per year, and it was steadily approaching the critical threshold of 120 million ha, which represents the boundary for farmland protection [1]. The saline soil is widely considered as a vital reserved land resource, as it possesses tremendous exploring potential. According to statistics, Jiangsu Province has a coastline stretching 954 km and an area of 0.7 million ha of saline soils. Northern Jiangsu Plain has a large area of concentrated and continuous coastal saline soils in China, with many ecological types and a high rate of siltation [2]. As a potential arable land resource, it is of great scientific significance to improve and make rational and efficient use of saline land to promote the development of agriculture [3]. While the saline soil usually demonstrated low productivity with high salt content, poor soil structure, low water permeability, and weak fertility, effective management and restoration of saline lands have been researched at home and abroad. In recent years, the use of water conservancy measures, such as fresh-water washing, buried pipe salt discharge, etc., has played a great role in improving the water and salt environments of saline soils in the costal tidal area [4]. However, due to the poor permeability, high groundwater level, and salinity of the costal soil, the salt reduction effect of water irrigation and drainage is not good, and it is easy to return salt. Adding exogenous amendments is also a convenient and economic option to improve the soil situation and enhance land productivity, as coastal saline soils are poorly structured and badly drained, and salts are slow to be exported with drainage.

Biochar is a new type of soil amendment, known as “black gold” in international academics. Biochar is a carbon-rich material, which is formed by the cracking of biomass under high temperatures and anoxic conditions [5]. Usually, common biochar feedstocks include lignocellulose from agricultural and forest wastes, as well as sewage sludge and organic waste. The common biochar selected for previous studies is generally alkaline in nature; therefore, its acidification is considered in the process of saline soil improvement to decrease the pH of biochar at the preparation stage or before addition, and using the obtained acid-modified biochar for the improvement and restoration of saline soils. Moreover, common biochar is treated with acid solutions to increase surface area, enrich pore structure, and increase oxygen content and hydrophilicity [6,7].

Many researchers have found that biochar has significant advantages in terms of improving soil structure, increasing soil nutrients, and ensuring better quality and increased plant yields when added to soil as an organic amendment [8,9,10,11]. Biochar reduces soil bulk density, increases porosity, and improves particle size distribution because of its porous structure and low bulk density. Biochar has a direct or indirect effect on soil infiltration processes, thereby increasing soil water content and permeability [12,13]. Cui et al. determined that biochar improved the properties of saline soils and reduced soil salt concentration [14]. Biochar has many functional groups and a high specific surface area, which improves the exchange of cations, ion exchange, and discharge of sodium ions in saline soils, and reduces the sodium adsorption ratio [15]. At the same time, biochar has a large number of nutrients and releases them slowly, thus improving soil fertility [16]. Biochar harmonizes soil water, fertilizer, gas, and heat factors, thus improving the growing environment and promoting plant growth. Xu reported that 3 t/ha biochar ensured stable maize yields at 30% nutrient reduction, and 3, 5, and 7 t/ha biochar significantly increased maize yields at adequate nutrient levels [17]. Guo reported that a biochar addition of 20 t/ha with 90% irrigation was most beneficial to stable and high yields of spring wheat in the northern border irrigation areas from field trials and model simulations [18]. The effects of biochar on crop growth and yield increase were particularly evident in soils with environmental stress conditions or low fertility.

Comprehensively, the effects of common biochar on plant growth have been widely reported; however, the effect on plants in saline soils is inconclusive. Furthermore, biochar is mainly alkaline, and there are few results available for the potential of acid-modified biochar to improve plant yield and quality in poorly structured, low-fertility, coastal tidal saline soils. Thus, we conducted an experiment (1) to investigate the effects of common biochar and acid-modified biochar on the growth and yield of spinach in coastal saline soils, (2) to clarify the impact of both kinds of biochar on the photosynthetic characteristics and quality of spinach, and (3) to discuss the comprehensive performance of spinach under each treatment based on IBRv2 and propose a suitable biochar addition plan for improving the productivity of coastal saline soils.

## 2. Results

### 2.1. Spinach Growth Conditions

**Figure 1:**
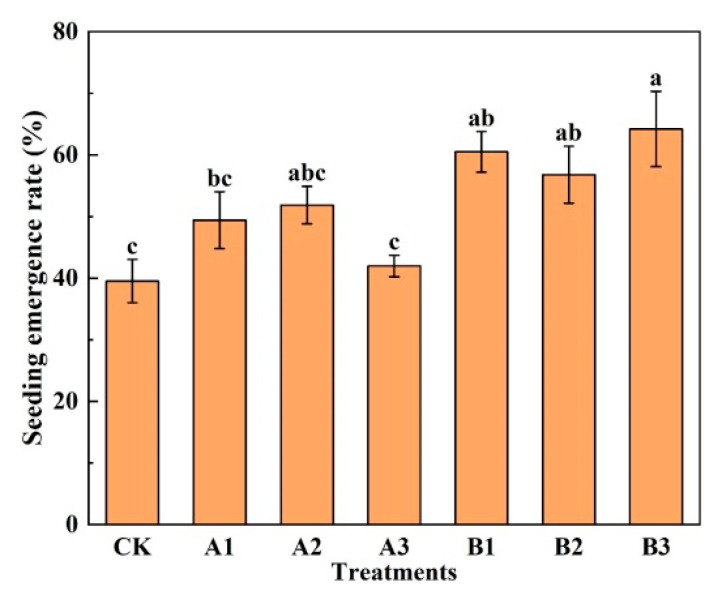
Seeding emergence rate of spinach under treatments. Notes: The different lowercase letters indicate significant difference (at *p* < 0.05) according to Duncan’s test. “CK” is the packed soil without biochar addition; “A1”, “A2”, and “A3” are the treatments of packed soil mixed with common biochar at the rates of 2%, 4%, and 8%, respectively; and “B1”, “B2”, and “B3” are the treatments of packed soil mixed with acid-modified biochar at the rates of 2%, 4%, and 8%, respectively.

The seeding emergence rate, which reflects the ability of seeds to withstand adversity, is an important index for plant growth. As can be seen in Figure 1, biochar addition improved the emergence of spinach seedlings. Compared with CK, the emergence rate of spinach was increased by 7.41–11.11% by adding common biochar, while there is no significant difference. The emergence rate under B1, B2, and B3 was significantly increased by 53.13%, 3.75%, and 62.50%, respectively. In the common biochar group, it was found that with the increase in the common biochar addition rate, the emergence of spinach increased first and then decreased, which indicated that it was not suitable to add excess common biochar. In the acid-modified biochar group, it was found that the emergence rate showed a trend of decreasing first and then increasing with the increase in the acid-modified biochar amount. It is noted that the acid-modified biochar at high additions had the most favorable effect on the emergence rate of spinach in saline soil.

**Figure 2:**
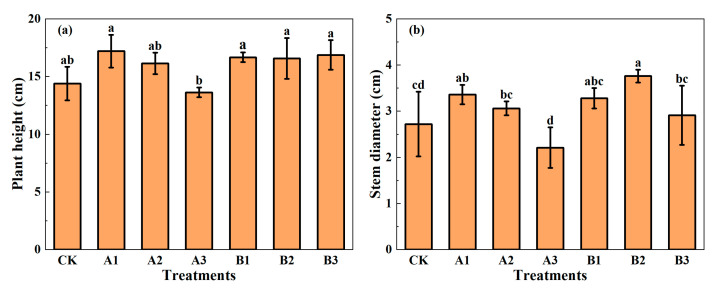
Plant height (**a**) and stem diameter (**b**) of spinach under treatments. Notes: The different lowercase letters indicate significant difference (at *p* < 0.05) according to Duncan’s test. “CK” is the packed soil without biochar addition; “A1”, “A2”, and “A3” are the treatments of packed soil mixed with common biochar at the rates of 2%, 4%, and 8%, respectively; and “B1”, “B2”, and “B3” are the treatments of packed soil mixed with acid-modified biochar at the rates of 2%, 4%, and 8%, respectively.

Plant height and stem diameter are important indicators to measure whether spinach grew well. It can be seen from Figure 2 that the addition of biochar increased the plant height and stem diameter of spinach, except A3. The plant height and stem diameter were significantly lower in A3 than those in A1, indicating that the addition of a high amount of common biochar was not conducive to plant growth. In the group of common biochar, the plant height and stem diameter of spinach decreased with the increase in biochar addition. In particular, A1 showed the best performance; compared to CK, the plant height and stem diameter increased by 15.18% and 23.67%, respectively. In the acid-modified biochar group, there was little difference in spinach plant height, but all were significantly higher than those under CK. The stem diameter of spinach increased first and then decreased with the increase in acid-modified biochar addition. The stem diameter of B2 was significantly higher than that of CK, while the stem diameter of B3 was significantly lower than that of B2. B2 showed the best performance in the acid-modified biochar group; compared to CK, the plant height and stem diameter of spinach increased by 10.94% and 38.38% respectively. The results showed that only B2 increased both the plant height and stem diameter significantly, indicating that B2 played the best role in improving both indicators.

### 2.2. The Fresh Weight and Dry Matter

**Figure 3:**
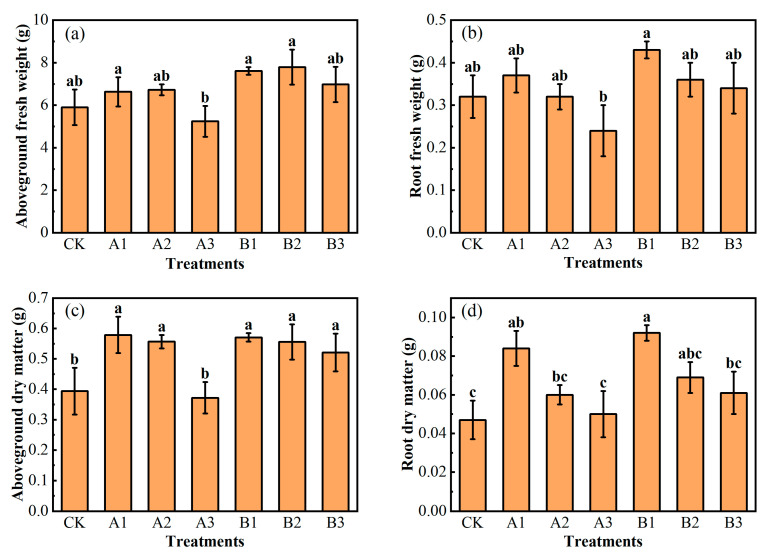
Aboveground fresh weight (**a**), root fresh weight (**b**), aboveground dry matter (**c**), and root dry matter (**d**) of spinach under treatments. Notes: The different lowercase letters indicate significant difference (at *p* < 0.05) according to Duncan’s test. “CK” is the packed soil without biochar addition; “A1”, “A2”, and “A3” are the treatments of packed soil mixed with common biochar at the rates of 2%, 4%, and 8%, respectively; and “B1”, “B2”, and “B3” are the treatments of packed soil mixed with acid-modified biochar at the rates of 2%, 4%, and 8%, respectively.

The aboveground fresh weight of all spinach showed a trend of decrease after the first increase, with the increase in both kinds of biochar addition. The aboveground fresh weight under A3 was significantly lower than that of A1, and even lower than that of CK. The addition of acid-modified biochar increased the aboveground fresh weight of spinach better than common biochar, but the difference was not significant. In the acid-modified biochar group, B2 had the highest aboveground fresh weight, but B3 obtained the lowest aboveground fresh weight (Figure 3a). The root fresh weight of spinach decreased gradually with the increase in biochar addition. All treatments had higher root fresh weight than CK, except A3, which was even lower than CK. The root fresh weight of the acid-modified biochar was greater than that of the common biochar with the same addition (Figure 3b). The aboveground dry matter of spinach decreased gradually with the increase in biochar addition All treatments increased the aboveground dry matter of spinach significantly, except A3. In detail, compared to CK, A1, A2, B1, B2, and B3 increased by 47.15%, 41.55%, 44.88%, 41,14%, and 32.40%, respectively (Figure 3c). The addition of biochar increased the root dry matter of spinach. The higher the biochar addition, the lower the root dry matter was. Compared to CK, the root dry matter of A1 and B1 were significantly improved by 80.14% and 97.14%, respectively (Figure 3d). The four graphs as a whole show that the high addition of biochar was not as effective in improving spinach growth as the low-to-medium additive levels of biochar. In particular, the high addition of common biochar had somewhat of an inhibiting effect. The results indicated that the appropriate addition of common biochar and acid-modified biochar was beneficial to accumulate aboveground dry matter and promote root growth. In summary, the addition of 2% of acid-modified biochar played a better role in increasing spinach biomass.

**Figure 4:**
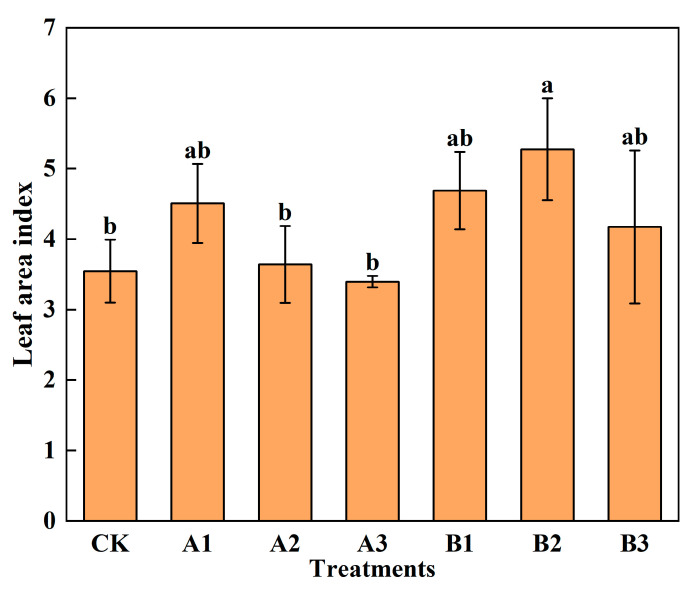
Leaf area index of spinach under treatments. Notes: The different lowercase letters indicate significant difference (at *p* < 0.05) according to Duncan’s test. “CK” is the packed soil without biochar addition; “A1”, “A2”, and “A3” are the treatments of packed soil mixed with common biochar at the rates of 2%, 4%, and 8%, respectively; and “B1”, “B2”, and “B3” are the treatments of packed soil mixed with acid-modified biochar at the rates of 2%, 4%, and 8%, respectively.

The leaf area index (LAI) is a key dynamic parameter that describes plant structure characteristics. It quantifies the total amount of leaf area in the canopy and impacts the growth status of spinach [19]. The LAI of spinach in Figure 4 showed a decreasing trend with the increase in the addition of common biochar, and the leaf area index of A3 was lower than that of CK. It indicated that the addition of biochar increased the leaf area index of spinach, but above the limit, the leaf area index decreased. The leaf area index of spinach increased first and then decreased with the increase in acid-modified biochar. B2 increased the leaf area index of spinach significantly, but other treatments did not affect the index significantly. The leaf area index of acid-modified biochar was higher than that of common biochar for the same amount of addition.

### 2.3. The Ratio of Root to Shoot and Healthy Strong Seeding Index

**Figure 5:**
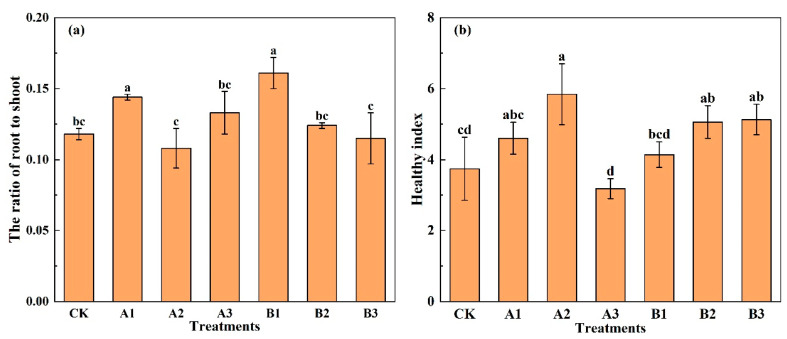
The ratio of root to shoot (**a**) and healthy index (**b**) of spinach under treatments. Notes: The different lowercase letters indicate significant difference (at *p* < 0.05) according to Duncan’s test. “CK” is the packed soil without biochar addition; “A1”, “A2”, and “A3” are the treatments of packed soil mixed with common biochar at the rates of 2%, 4%, and 8%, respectively; and “B1”, “B2”, and “B3” are the treatments of packed soil mixed with acid-modified biochar at the rates of 2%, 4%, and 8%, respectively.

As we can see in Figure 5a, the low addition of biochar increased the ratio of root to shoot (*R_s_*) of spinach clearly, while the medium-to-high addition of biochar performed poorly. The ranking order of the *R_s_* of spinach under different treatments was B1 > A1 > A3 > B2 > CK > B3 > A2. The *R_s_* of spinach under Al and B1 was greater than that of CK significantly, with an increase of 22.86% and 37.27%, respectively. The results showed that the distribution of aboveground photosynthetic products to the root system was increased under low biochar addition. The *R_s_* of spinach under A2 and B3 was smaller than that of CK, with a decrease of 8.11% and 2.09%, respectively.

As it can be seen in Figure 5b, biochar addition improved the healthy index (*HI*) of spinach, except A3. Compared to CK, A2 significantly increased the healthy index by 56.03%. The highest common biochar addition treatment (A3) obtained the lowest healthy index, which showed that common biochar utilization should have a threshold rate. With the increase in the acid-modified biochar addition, the healthy index improved, but there is no significant difference among B1, B2, and B3. Compared to CK, the *HI* of B2 and B3 increased significantly by 35.29% and 37.17%, respectively.

### 2.4. Spinach Yield

**Figure 6:**
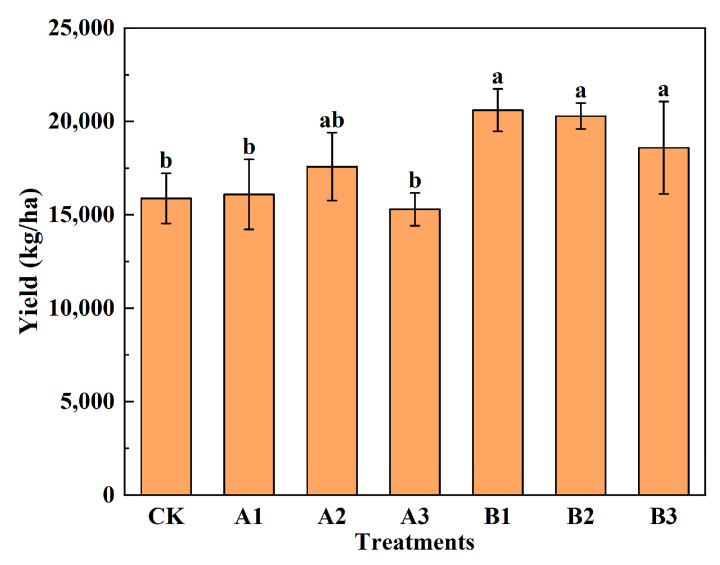
The yield of spinach under treatments. Notes: The different lowercase letters indicate significant difference (at *p* < 0.05) according to Duncan’s test. “CK” is the packed soil without biochar addition; “A1”, “A2”, and “A3” are the treatments of packed soil mixed with common biochar at the rates of 2%, 4%, and 8%, respectively; and “B1”, “B2”, and “B3” are the treatments of packed soil mixed with acid-modified biochar at the rates of 2%, 4%, and 8%, respectively.

It can be seen from Figure 6 that both kinds of biochar addition can effectively increase spinach yields, except A3. The yield of spinach increased first and then decreased with the increase in common biochar; however, it showed a decreasing trend with the increase in the acid-modified biochar. Compared to CK, the yield of spinach was increased by 10.7% under A2, while it reduced by 3.7% under A3. This indicated that the addition of common biochar exceeding the upper limit will lead to yield decline. Compared with CK and the common biochar group, acid-modified biochar significantly increased spinach yield, but there is no significant difference inside the group. The highest yield was obtained under B1, which was increased by 29.7%, compared to CK. This indicated that lower acid-modified biochar addition had a good effect on the yield increase of spinach; however, the addition of more than 8% will reduce the yield.

### 2.5. Total Water Consumption and Water Productivity

As noted in Table 1, the trend in total irrigation and *ET* data across the trials was consistent. During spinach growth, *ET* increased in all treatments, except for A1, where *ET* was lower than CK. The *ET* of spinach treated with acid-modified biochar was higher than that with common biochar, except for A2. In our trial, *ET* included transpiration evaporation, as well as spinach growth water consumption. WP reflects the agricultural output achieved per unit of water resources consumed. Biochar increased the WP of spinach in the descending order of B2, B1, B3, A2, A1, CK, A3. Clearly, biochar enhanced the WP of spinach, except for A3. The WP of both types of biochar exhibited an initial increase, followed by a decrease as the addition rates rose. Excessive addition rates were likely to have diminished WP, a result that was already demonstrated by A3. The WP of acid-modified biochar was higher than that of common biochar, particularly in the cases of B1 and B2, where the increments were significantly higher than CK, with values of 25.50% and 25.85%, respectively.

### 2.6. Spinach Photosynthetic Characteristics

Plant photosynthetic indices furnish important insights into the physiological and metabolic health of plants, encompassing a range of critical parameters, including net photosynthesis rate, intercellular carbon dioxide concentration, transpiration rate, and stomatal conductance.

**Figure 7:**
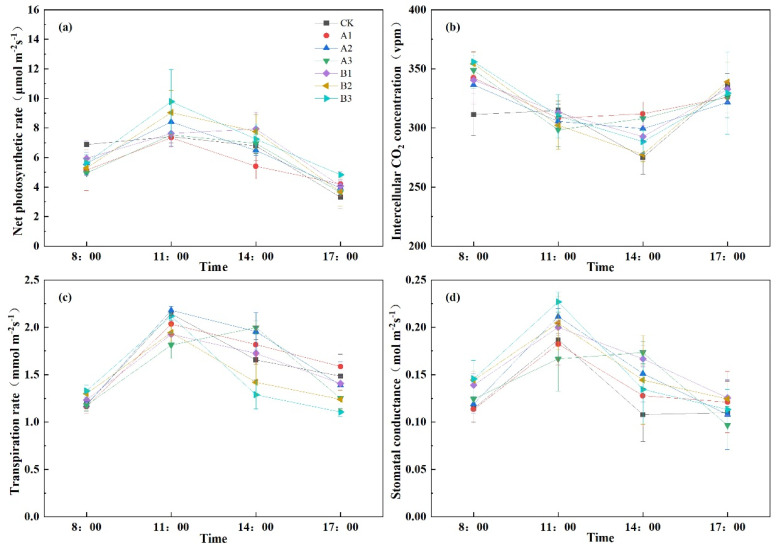
The photosynthetic characteristics of spinach under treatments. Notes: (**a**) is net photosynthetic rate, (**b**) is intercellular carbon dioxide concentration, (**c**) is spinach’s transpiration rate and (**d**) is spinach’s stomatal conductance. The different lowercase letters indicate significant difference (at *p* < 0.05) according to Duncan’s test. “CK” is the packed soil without biochar addition; “A1”, “A2”, and “A3” are the treatments of packed soil mixed with common biochar at the rates of 2%, 4%, and 8%, respectively; and “B1”, “B2”, and “B3” are the treatments of packed soil mixed with acid-modified biochar at the rates of 2%, 4%, and 8%, respectively.

The primary objective of augmenting plant productivity is centered on elevating the net photosynthetic rate. It can be seen from Figure 7a that the variations in daily net photosynthetic rates among the different treatments displayed a coherent pattern, conforming to an initial increase, followed by a subsequent decrease. At 8 AM, when photosynthetically active radiation was relatively diminished, spinach exhibited a correspondingly reduced net photosynthetic rate. Interestingly, biochar restrained plant photosynthesis. In contrast, by 11 AM, as photosynthetically active radiation intensified, spinach demonstrated a proportional enhancement in the net photosynthetic rate. Except for A1, all other interventions stimulate photosynthetic activity, with B3 significantly magnifying the net photosynthetic rate. At 2 PM, under elevated temperatures, common biochar exhibited a degree of inhibition on spinach photosynthesis, while acid-modified biochar still augmented the net photosynthetic rate. These collective observations underscore that, under abundant illumination, the incorporation of acid-modified biochar effectively heightened spinach’s net photosynthetic rate.

In Figure 7b, spinach exhibited a pattern of declining intercellular carbon dioxide concentration, followed by subsequent elevation. At 8 AM and 2 PM, biochar induced a relative increase in intercellular carbon dioxide concentration compared to CK. In contrast, at 11 AM, biochar led to a relative reduction. By 5 PM, A2 displayed a 0.79% increase relative to CK, while other treatments showed decreases.

Influenced by environmental factors, spinach’s transpiration rate followed a pattern of increase firstly and then decrease in Figure 7c. Transpiration was minimal at 8 AM, peaked at 11 AM, and gradually declined thereafter. At 8 AM, B2 and B3 exhibited significantly higher transpiration rates than CK, with increases of 9.54% and 11.88%, respectively. By 11 AM, only A2 surpassed CK in transpiration, while A3, Bl, and B2 displayed significantly lower rates. At 2 PM, B2 and B3 exhibited reduced transpiration compared to CK. Subsequently, at 5 PM, A2, A3, and B3 significantly exhibited lower transpiration rates than CK.

It can be seen from Figure 7d that stomatal conductance and the net photosynthetic rate followed a consistent pattern, initially increasing and then decreasing. Compared to CK, acid-modified biochar significantly enhanced spinach stomatal conductance at 8 AM. By 11 AM, with increasing light intensity, stomatal conductance rose, except for A1 and A3, which were lower than that of CK. From 2 PM to 5 PM, stomatal conductance declined, yet acid-modified biochar remained effective in promoting increased conductance.

### 2.7. Spinach Qualities

Soluble sugars and proteins in plants are energy sources for plant growth and development and play an important role in maintaining plant growth and metabolism and ensuring the plant survival rate. The contents of soluble sugar and protein in spinach were detected after harvest. The results are shown in Table 2. When common biochar was added, the soluble sugars decrease at higher additions, while the opposite was true for the acid-modified biochar. Compared with CK, the soluble sugar content of the edible part of spinach was significantly increased under A1, B2, and B3. Biochar increased the soluble protein content of spinach. The soluble protein content tends to increase and then decrease as the amount added increases. Biochar increased the soluble protein content of spinach. As the addition of biochar added increased, the soluble protein content tended to increase and then decrease. Compared to CK, the addition of 4% and 8% biochar increased the soluble protein content of spinach significantly.

Nitrite has been proven to have adverse effects on health, and it is essential to reduce the nitrite content in vegetables. Nitrite content is an important indicator of spinach quality. Biochar affected the nitrite content of the edible part of the spinach, reducing its content. The nitrite content in the edible portion of spinach was significantly reduced under B2, and a reduction ratio of 31.85% was achieved, compared to CK.

### 2.8. The IBRv2 Calculation

**Figure 8:**
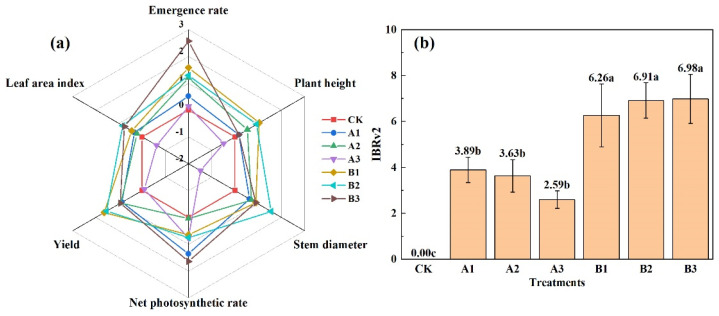
Associated radar plots (**a**) and IBRv2 values (**b**) under different biochar types and additions. Notes: “CK” is the packed soil without biochar addition; “A1”, “A2”, and “A3” are the treatments of packed soil mixed with common biochar at the rates of 2%, 4%, and 8%, respectively; and “B1”, “B2”, and “B3” are the treatments of packed soil mixed with acid-modified biochar at the rates of 2%, 4%, and 8%, respectively.

Based on the above results, six plant indexes with significant changes in seedling emergence, plant height, stem diameter, leaf area index, yield, and net photosynthetic rate were selected. As seen in Figure 8, the seedling emergence rate was activated and the rest of the indicators were inhibited in A3. This indicated that excess addition of common biochar could promote the emergence of spinach, but was not conducive to the growth of spinach in the middle and later growth stages. A1 and A2 had activation effects in the six indicators, and each activated indicator was close to 0; the value was low, and the activated effect was not obvious. The acid-modified biochar treatments had activation effects on seedling emergence, plant height, stem diameter, leaf area index, yield, and net photosynthetic rate. The activated indicator was higher than that of the common biochar treatments, and the activated effect was more obvious.

When calculated by integrated biological response version 2, the IBRV2 values under acid-modified biochar treatments were significantly higher than those under common biochar treatments (*p* < 0.05). However, there is no significant difference among acid-modified biochar treatments. The IBRv2 value of acid-modified biochar demonstrated a substantial increase in comparison to CK and common biochar. This enhancement was underscored by the activation effects observed across all assessed indicators. This suggested a pronounced amelioration of spinach growth within saline-alkali soil upon the incorporation of acid-modified biochar.

## 3. Materials and Methods

### 3.1. Tested Soil, Materials, and Acid-Modified Biochar Preparation

The tested soil was collected from coastal areas located in Dongtai City, Jiangsu Province. The detailed soil properties are listed in Table 3. The soil particle size composition was measured using a Malvern laser particle size analyzer (MS–3000). The proportion of clay (particle size < 0.002 mm), silt (particle size 0.002–0.02 mm), and sand (particle size 0.02–2 mm) was 7.44%, 49.10%, and 43.46%, respectively, and the test soil texture can be classified as a silty, sandy loam, according to the International Soil Classification System [20].

The common biochar used in this experiment was obtained from Henan YuZhongAo Agricultural Technology Company. The original material was wheat straw and was pyrolyzed at 550–600 °C for 4–6 h. The biochar was in granular form, with an initial particle size of 2–4 mm and a bulk density of 0.19 g/cm^3^. The specific surface area was 9 m^2^/g, the total porosity was 67.03%, and the pH value was 10.24. The biochar was ground and sieved via a 2 mm stainless steel sieve. 

Acid-modified biochar preparation: The common biochar was placed into a container, and phosphoric acid solution with a mass fraction of 25% was added into it at a ratio of 1:2, shaken thoroughly, mixed well, and left to stand for more than 30 min. After complete acidification, the solid–liquid separation and washing of the biochar with ultrapure water were carried out until the pH of the eluate was constant, then natural air drying, grinding, and passing through a 2 mm sieve to obtain acid-modified biochar. The specific surface area of acid-modified biochar was 51.67 m^2^/g, and the pH value was 3.42.

The pots are plastic buckets, with a diameter of 21 cm and a height of 25 cm. Some small holes were drilled in the bottom for free drainage. A layer of gauze was laid down before filling with soil to prevent blocking the drainage holes. Prior to the experiment, the collected soil was air-dried and sieved via a 2 mm stainless steel sieve. We calculated the amount of biochar and soil before filling the soil and mixed them thoroughly to minimize variability. The soil was packed into pots, with every 5 cm layer achieving a natural bulk density of around 1.35 g/cm^3^. The total soil depth of the pot was 20 cm, with a 5 cm gap for retaining water. Each layer was lightly raked before packing the next layer to minimize discontinuities between layers. To prevent the upper fine particles from plugging the apertures, two layers of geotextile were set at the bottom before packing the soil.

Prior to the experiment, the pots were irrigated with 3 L of distilled water, according to the saturated water content of the soil. Nine sowing spots with a space of 5 cm were selected in a pot, and three spinach seeds were sowed. The spinach was sown on 4 May 2020, and only one spinach plant was kept at each point after the seedlings emerged on 11 May 2020. During the trial, the pots were irrigated to maintain suitable soil moisture content (80% of field capacity). The pots were weighed by an electronic scale (the accuracy is 0.1 g), and the differences in the weighing were the amount of water required for irrigation. The soil was fertilized at a rate equivalent to 65.7 g/m^2^ with a compound fertilizer as the base fertilizer, of which the percentage of *N-P-K* was 15:15:15, and the nutrient total was ≥45%. 

### 3.2. Experimental Site and Design

The experiment was carried out in a greenhouse at the South Road Campus of Yangzhou University (119°25′17″ E, 32°22′33″ N) in southern China from May to July 2020. The site is classified as a humid subtropical climate (Köppen Climate), which is a typical climate of eastern China. We maintained the soil moisture content at around 80% of the field capacity during planting and avoided natural rainfall and strong sunlight.

The study evaluated seven treatments with three replicates; the pot only with packed soil, without biochar addition, was used as a control (CK) to simulate the standard local practice. The other six treatments were the packed soil with the addition of (1) 2% common biochar (A1), (2) 4% common biochar (A2), (3) 8% common biochar (A3), (4) 2% acid-modified biochar (B1), (5) 4% acid-modified biochar (B2), and (6) 8% acid-modified biochar (B3). The above biochar additions of 2%, 4%, and 8% were all calculated based on the dry soil mass and evenly mixed with soil.

### 3.3. Observation and Measurement

Prior to the experiment, 27 spinach seeds were meticulously sown in each pot, and the emergence rate was recorded on the 7th day. During the growth stage, a steel tap (the accuracy is 0.01 m) was used to measure the plant height from the ground to the highest point of a natural extension of all leaves, and the stem diameter was measured by a digital vernier caliper with an accuracy of 0.01 m. The spinach, except for the roots, was removed from the soil at physiological maturity and then weighed as the aboveground fresh weight. The roots were removed from the soil completely, the remaining soil rinsed off, any excess moisture dried off, and then they were weighed as the root fresh weight. Dry matter was measured by drying the aboveground part and the root part at 105 °C for 30 min and then oven drying at 75 °C until a constant weight. At harvest time, we measured the fresh weight of the whole spinach plant in each pot to obtain the harvest yield per pot and then converted it into spinach’s yield with the unit of kg/ha by using the pot area.

The photosynthetic characteristics were measured by the LCpro-SD fully automated portable photosynthesis analyzer (ADC, UK) on a sunny day prior to harvest. At four specific time points (8:00 AM, 11:00 AM, 2:00 PM, and 5:00 PM), three moderately developed spinach plants were deliberately selected from each pot to conduct the photosynthesis measurements meticulously.

The leaf area index (LAI) was calculated as follows by measuring the length and width of a leaf,

(1) $$\mathrm{LAI}=K\times \frac{\rho \sum_{i=1}^{n}(L_{i}\times B_{i})}{A}$$

where *K* was the plant simulate coefficient, which is 0.75 for maize; *L_i_* and *B_i_* were the length and width of the leaf (cm), respectively; and *A* was the land area occupied by the plant (cm^2^). 

The ratio of root to shoot (*R_s_)* was calculated as follows, based on the conversion of shoot and root dry matter,

(2) $$R_{s}=\frac{R}{T}$$

where *R* was the root dry matter, and *T* was the aboveground dry matter.

The healthy index (*HI*) was calculated as follows,

(3) $$HI=\left(\frac{S}{H}+R_{S}\right)\times h$$

where *S* was the stem diameter (cm), *H* was the plant height (cm), and *h* was the dry matter of the plant (g).

The total water consumption (*ET*) was calculated as follows, based on the irrigation water,

(4) ET=P+I+ΔW−R−D

where *P* was the precipitation, *I* was the irrigation water (mm), ΔW was the soil moisture variation (mm), *R* was the surface runoff, and *D* was the deep percolation (mm).

The trial environment was sheltered from rain, and no surface runoff was generated, so the equation can be simplified as the following,

(5) ET=I+ΔW−D

The water productivity (WP) was calculated as follows,

(6) $$\mathrm{WP}=\frac{Y}{ET}$$

where *Y* was the yield of spinach (kg/ha).

The value of integrated biological response version 2 (IBRv2), proposed by Sanchez et al., is applied to assess the overall biological response to changes in soil and plant conditions resulting from different agricultural management practices [21]. It allows researchers to quantify and compare the impact of various soil improvement measures on soil health and agricultural productivity. The value of IBRv2 was calculated as follows, using the main indexes, soil, and plant.

(7) $$\mathrm{IBRv}2=\sum_{i=1}^{n}\left|A_{i}\right|$$

(8) $$A_{i}=\left(\frac{log\frac{x_{i}}{x_{0}}-\mu }{\sigma }\right)-Z_{0}$$

The influencing factors with a strong correlation were selected, and the IBRv2 index was calculated, and higher IBRv2 values indicated larger deviations for all selected factors with regard to the chosen reference level.

### 3.4. Data Analysis and Statistics

Microsoft Excel 2010 (Microsoft Corporation, Redmond, WA, USA, 2010) was used for data calculating and processing. The significant differences among treatments were determined by Duncan’s test, using the SPSS 20.0 statistical program (International Business Machines Corporation, Armonk, NY, USA,2011). The figures were plotted and exported by Origin 8.0 (OriginLab Corporation, Northampton, MA, USA, 2007).

## 4. Discussion

Suitable soil conditions are essential for the germination and growth of plants. Ali et al. revealed that biochar addition increased seed emergence and shortened the mean emergence time in days [22]. Similarly, spinach seedling emergence was increased by the addition of biochar, with a significant positive effect of acid-modified biochar in this trial. The possible reason for better seed emergence is more water content retained around seeds by biochar, as reported by Claudia et al. [23]. The low moisture level in coastal saline soils reduces seed emergence; adding biochar into the soil can overcome this weakness, which enhances the water-holding capacity in a growing medium, which ultimately increases seed emergence. Similar to previous studies [24,25], the addition of biochar to coastal saline soils promoted the plant height and stem diameter in this trial. It may be explained that biochar functioned as a nutrient retarder, prolonged the soil fertilizer availability, adsorbed and held soil nutrients, and activated soil microorganisms [26]. Also, it may be that biochar carried its mineral nutrients, which were released slowly, thus making the soil fertility condition promote growth better in some way [27]. Here, it is important to note that too much common biochar was detrimental to plant growth properties. The possible reason is that biochar can reduce the soluble salt content of the soil, but excessive biochar addition will inversely increase the soil salinity [28,29]. Biochar had a diluting effect on the soluble salt content of soil, mainly because the soluble salt content of biochar is lower than that of soil. In addition, biochar adsorbed salts from the soil [30], thus reducing the soil soluble salt content. Likewise, the performance of biochar on the LAI of spinach may be the same reason. It was found that the dry matter quality and yield of spinach were improved by the addition of biochar with fertilizer in our trial. It is probably because the appropriate biochar addition improved the absorption capacity of the root system and the supply of nutrients to the aboveground parts, which is important for the absorption and transport of water and nutrients in the soil. Wheat straw biochar was chosen as an organic amendment to the soil, with a high ash content and a nutrient supplement to soil with relatively low fertility. Biochar led to an increase in the WP of spinach, indicating an increase in the amount of spinach yield produced per unit mass of water consumed. It was possible that biochar effectively reduced the saline soil bulk density [31]. Filling the pots according to the experimental design soil bulk density improved the compactness of the soil, allowing the small dispersed particles in the soil to agglomerate and form large agglomerates, increasing the number of large pores, reducing the capillary force of rising soil water, and promoting water infiltration. At the same time, biochar lowered the pH of saline soils and reduced the rate of salt accumulation, which inhibits the accumulation of salts in the surface layer of the soil effectively [29]. To sum up, biochar was reported to improve the soil’s water and salt environments, thus increasing the yield of spinach.

The extent of plants’ photosynthetic capability directly relates to the growth status of both their aboveground and root components. Acid-modified biochar notably enhanced the net photosynthetic rate, stomatal conductance, and transpiration rate, effectively bolstering the plant photosynthetic performance and ensuring ample support for efficient photosynthesis. The increased stomatal conductance and transpiration rate supply favorable conditions for enhanced water and nutrients uptake by the plant’s root system, as well as the efficient transport of synthesized nutrients to the aboveground portions.

Plants under stress actively accumulate osmoregulatory substances to reduce the cellular water potential to maintain normal expansion pressure and increase plant resilience. It is important to increase the content of soluble sugars and soluble proteins, both of which are important osmoregulatory substances, to promote the plant’s ability to regulate and adapt to adverse conditions [32]. The low and medium addition rate of acid-modified biochar significantly increased the soluble sugar and the soluble protein content of spinach. It was mainly because the acid-modified biochar alleviated the effects of saline soil on plant respiration and photosynthesis, up-regulated enzyme activities related to sugar and nitrogen metabolism, as well as enhanced the salt resistance of the plant [33]. Biochar promoted rapid nutrient uptake by plant roots from the growing environment, reduced the amount of nitrogen left in the soil, speeded up the processes of nitrate conversion and uptake in the plant, and improved the utilization of fertilizers.

Furthermore, the acid-modified biochar had a more favorable effect on the plant dry matter quantity and yield at the same addition level compared to common biochar in this trial. This was probably due to the use of phosphoric acid to remove metals from the surface of biochar and impurities from the its pores, optimizing the physicochemical properties of the biochar, which increased the pore structure and specific surface area of the biochar [34]. Acid-modified biochar absorbed more nutrient ions with larger pores and more internal pores, which were released slowly during plant production, reducing soil nutrient loss. Common biochar is generally alkaline, but the use of phosphoric acid modification reduced the pH of the biochar itself. When acid-modified biochar is added to saline soils, it neutralizes a certain amount of alkalinity and regulates the pH of the soil more effectively.

## 5. Conclusions

In conclusion, biochar effectively promoted the emergence rate, growth, and yield, as well as the quality, of spinach, and it had the potential to elevate spinach’s photosynthetic characteristics; these effects were particularly pronounced when adding moderate-to-high levels of acid-modified biochar. However, the high addition of common biochar was to some extent detrimental to plant growth and quality. Taking economic factors into account, applying acid-modified biochar at the rate of 4% is a suitable option to ensure the increasing yield and quality of spinach in costal saline soils.

## Figures and Tables

**Table 1:** The total irrigation water, *ET*, and WP under treatments.

Treatment	Total Irrigation Water Amount (mm)	*ET* (mm)	WP (kg/m^3^)
CK	64.95 ± 1.94 ^ab^	66.40 ± 1.87 ^ab^	239.52 ± 28.09 ^b^
A1	62.83 ± 1.86 ^b^	64.30 ± 1.72 ^b^	250.85 ± 21.04 ^ab^
A2	66.18 ± 3.53 ^a^	67.78 ± 3.43 ^a^	260.36 ± 20.66 ^ab^
A3	65.57 ± 0.56 ^ab^	66.95 ± 0.50 ^ab^	228.53 ± 16.98 ^b^
B1	66.69 ± 2.52 ^a^	68.56 ± 2.43 ^a^	301.12 ± 18.52 ^a^
B2	65.47 ± 3.20 ^ab^	67.31 ± 3.27 ^ab^	301.44 ± 3.45 ^a^
B3	66.74 ± 1.22 ^a^	68.43 ± 1.07 ^a^	271.89 ± 26.67 ^ab^

Notes: Different lowercase letters in each column indicate significant differences between treatments (*p* < 0.05; Duncan’s test). The value after “±” is the standard deviation of replicates. *ET* is the total water consumption; WP is the water productivity. “CK” is the packed soil without biochar addition; “A1”, “A2”, and “A3” are the treatments of packed soil mixed with common biochar at the rates of 2%, 4%, and 8%, respectively; and “B1”, “B2”, and “B3” are the treatments of packed soil mixed with acid-modified biochar at the rates of 2%, 4%, and 8%, respectively.

**Table 2:** The nutrients quality of spinach under treatments.

Treatment	Soluble Sugar (mg/g)	Soluble Protein (mg/g)	Nitrite (mg/kg)
CK	8.27 ± 0.30 ^c^	1.72 ± 0.13 ^c^	1.50 ± 0.21 ^a^
A1	9.86 ± 0.34 ^b^	1.87 ± 0.10 ^bc^	1.12 ± 0.08 ^ab^
A2	9.35 ± 0.77 ^bc^	2.35 ± 0.08 ^a^	1.23 ± 0.21 ^ab^
A3	6.96 ± 0.24 ^d^	1.93 ± 0.04 ^b^	1.25 ± 0.14 ^ab^
B1	9.72 ± 0.45 ^bc^	1.80 ± 0.02 ^bc^	1.17 ± 0.15 ^ab^
B2	10.23 ± 0.31 ^b^	2.20 ± 0.03 ^a^	1.02 ± 0.13 ^b^
B3	13.92 ± 0.71 ^a^	1.93 ± 0.04 ^b^	1.19 ± 0.23 ^ab^

Notes: Different lowercase letters in each column indicate significant differences between treatments (*p* < 0.05; Duncan’s test). The value after “±” is the standard deviation of replicates. “CK” is the packed soil without biochar addition; “A1”, “A2”, and “A3” are the treatments of packed soil mixed with common biochar at the rates of 2%, 4%, and 8%, respectively; and “B1”, “B2”, and “B3” are the treatments of packed soil mixed with acid-modified biochar at the rates of 2%, 4%, and 8%, respectively.

**Table 3:** The properties of tested soil.

Soil Bulk Density (g/cm^3^)	Field Capacity (%)	Soil Organic Matter (g/kg)	Available Nitrogen (mg/kg)	Water-Soluble Organic Carbon (mg/kg)	pH	EC (mS/cm)
1.4	24.55	8.14 ± 0.07	15.66 ± 4.30	<0.5	7.96 ± 0.01	2.02 ± 0.02

Notes: the value after “±”represents the standard deviation of replicates.

## Data Availability

The data that support the findings of this study are available from the corresponding author [J.W.] upon reasonable request.
